# Phase-space studies of backscattering diffraction of defective Schrödinger cat states

**DOI:** 10.1038/s41598-021-90738-x

**Published:** 2021-06-02

**Authors:** Damian Kołaczek, Bartłomiej J. Spisak, Maciej Wołoszyn

**Affiliations:** Faculty of Physics and Applied Computer Science, AGH University of Science and Technology, al. Mickiewicza 30, 30-059 Kraków, Poland

**Keywords:** Matter waves and particle beams, Quantum mechanics, Theoretical physics

## Abstract

The coherent superposition of two well separated Gaussian wavepackets, with defects caused by their imperfect preparation, is considered within the phase-space approach based on the Wigner distribution function. This generic state is called the defective Schrödinger cat state due to this imperfection which significantly modifies the interference term. Propagation of this state in the phase space is described by the Moyal equation which is solved for the case of a dispersive medium with a Gaussian barrier in the above-barrier reflection regime. Formally, this regime constitutes conditions for backscattering diffraction phenomena. Dynamical quantumness and the degree of localization in the phase space of the considered state as a function of its imperfection are the subject of the performed analysis. The obtained results allow concluding that backscattering communication based on the defective Schrödinger cat states appears to be feasible with existing experimental capabilities.

## Introduction

The principle of superposition of states, which is an inherent feature of quantum theory, constitutes the fact that the sum of any two or more states also describes a possible state of an isolated system^1^. In this spirit, the linear superposition of two states can be defined by the formula

1 $$\begin{aligned} |\psi (t)\rangle = A \left\{ \sqrt{1-\beta }|\phi _1(t) \rangle + \sqrt{\beta } e^{i\vartheta } |\phi _2(t) \rangle \right\} , \end{aligned}$$

where $\left| \phi _1(t)\right\rangle$ and $\left| \phi _2(t)\right\rangle$ are eigenstates of some observable, *A* is the normalization factor, parameter $\beta$ controls the amplitude ratio of the states, and $\vartheta$ is the relative phase between them. This principle has been tested in numerous quantum interference experiments involving photons, electrons, neutrons, atoms or molecules, and currently there is no doubt that the superposition of such states exists at least at the microscopic scale^2^. Apart from this, in recent years more and more attention has been devoted to the experimental verification of basic ideas and predictions of quantum theory in order to get a better insight into the quantum properties of light and matter^3^. These experimental activities create new possibilities for generating, detecting, and measuring quantum states as well as controlling and steering them. The latter two are especially important for applications, and they are the subject of quantum engineering which is usually based on optical or solid-state solutions. One of the fundamental issues for applications in quantum information processing, including quantum computation, cryptography, metrology and teleportation, is the generation of highly non-classical states. A good example of such a state is the coherent superposition of two or more fairly well separated, distinguishable, localized states of a single system, which by analogy with the famous Schrödinger thought experiment is called the Schrödinger cat state^4–6^. Preparation of a macroscopic superposition of states in real systems is a difficult task because they are very sensitive to interaction with the environment, and require highly sophisticated methods to minimize the impact of processes caused by the dissipative influence of the environment. Otherwise, uncontrolled interactions of the system with the environment lead to a decay of the initially prepared macroscopic superposition of states, and as a result a statistical mixture of states is formed^7,8^. Nowadays, the non-classical states in the form of the Schrödinger cat states are created in systems such as laser-cooled trapped atoms and ions, photons in a microwave cavity, magnetic fluxes in SQUIDs, or nitrogen-vacancy centers in diamond mechanical resonators^9^. Moreover, effectively generated Schrödinger cat states can be used for studying complex systems on the basis of the coherent multidimensional spectroscopy^10^.

The possibility of creating non-classical states, e.g. the above mentioned Schrödinger cat states, raises natural questions about their dynamics in dispersive media and the accompanying effects. This problem is rarely analyzed in detail^11–14^, although it seems to be important for applications in quantum communications^15^ and problems related to a quantum state’s transmission or detection because of decoherence which destroys the quantum interference.

The aim of our work is to investigate the coherent dynamics of a defective superposition of two Gaussian wavepackets in the backscattering diffraction regime^16,17^ using the phase-space representation of the quantum theory^18–24^. The considered regime is determined by the above-barrier reflection process^1^ and it does not have a counterpart in the classical mechanics. Motivated by the non-classical character of this phenomenon we perform the phase-space analysis of this issue by exploring the coherent dynamics of the above-mentioned non-classical states in the backscattering diffraction regime. The probability of the above-barrier reflection is determined for non-classical states which differ by defectiveness. The barrier is modelled by a Gaussian function since it leads to results which do not differ substantially from results obtained when different soft-shaped narrow barriers are assumed. Additionally, as the Gaussian function is a commonly used model of a barrier or scattering center, our results may be easily compared with other studies of the coherent dynamics of quantum states^25,26^ or their mixtures^14^. However these two limiting cases explore properties of the tunnelling and reflection process below the barrier. On the other hand, the above-barrier tunnelling and reflection has been studied by Sokolovski^27^, and Petersen and Pollak^28^ in terms of the Gaussian wavepacket. Therefore our approach can be regarded as a generalisation of these results. Apart from that we directly investigate the dynamical aspects of the quantumness corresponding to this state as well as its degree of localization in the phase space using the appropriate entropic measure^29,30^. For these phase-space studies we have decided to choose the joint position-momentum distribution function in the form proposed by Wigner^31–34^ because its negativity in some regions of the phase space is regarded as the hallmark of non-classicality of the state^35–39^. Time-evolution of the Wigner distribution function (WDF) is governed by the Moyal equation of motion^40,41^ which describes, in the considered case, propagation of the constructed defective non-classical state in a one-dimensional dispersive medium with an obstacle modeled by the narrow Gaussian barrier. This model allows us to determine the above-barrier reflection regime and investigate the influence of the imperfection of the state preparation on backscattering diffraction. Additional aspect of this work is related to the application of the phase space formulation of quantum mechanics. This autonomous approach makes the quantum mechanics similar to statistical mechanics with ordinary *c*-functions which form the non-commutative algebra of observables on the phase space. This point of view permits a unified investigation of a variety of problems in classical and quantum systems and it yields deeper understanding of relations between the quantum and classical theories of dynamical systems.

Finally, it is also worth mentioning that one of the advantages of using the WDF is that it can be reconstructed from experimental data. This reconstruction allows one to visualize the WDF for different physical systems such as states of trapped light^42^, states of the microwave field^43^, molecular vibrational states, electrons and ions states^44–46^, thermal states^47,48^ or states of the spin systems^49^.

## Theoretical framework

### P$\hbar$ase space approach

In the phase-space formulation of quantum theory, a physical system is characterized by the Weyl symbol of the quantum-mechanical Hamiltonian, ${\hat{H}}({\hat{x}}, {\hat{p}})$, which is a Hermitian operator acting on the Hilbert space. Here we assume that the Hamiltonian is taken in the one-particle form, i.e. ${\hat{H}}={\hat{p}}^2/(2m)+U({\hat{x}})$, where *m* is the mass of a particle, $U({\hat{x}})$ is the potential energy operator, while ${\hat{x}}$ and ${\hat{p}}$ are the non-commuting quantum-mechanical operators of position and momentum respectively. The explicit form of the Weyl symbol of the Hamiltonian is given by the formula^20^

2 $$\begin{aligned} H_W(x,p) = \int {dX}\; \left\langle x+\frac{X}{2}\left| {\hat{H}}({\hat{x}}, {\hat{p}}) \right| x-\frac{X}{2}\right\rangle \exp \left( -\frac{ipX}{\hbar }\right) . \end{aligned}$$

In the same way, other Hermitian operators, ${\hat{\Omega }}\big ({\hat{x}}, {\hat{p}}\big )$, corresponding to dynamical variables, $\Omega (x, p)$, that characterize the system are represented by the appropriate Weyl symbols, $\Omega _W(x,p)$. In principle, the Weyl symbols of Hermitian operators can be regarded as real smooth functions acting on the phase space. These functions form a non-commutative algebra with respect to the Weyl–Groenewold product (star product) which is defined in the following way^50,51^,

3 $$\begin{aligned} *= \exp {\left[ \frac{i\hbar }{2} \left( \overleftarrow{\partial _x} \overrightarrow{\partial _p} - \overleftarrow{\partial _p} \overrightarrow{\partial _x} \right) \right] }, \end{aligned}$$

where the arrows indicate in which direction the derivatives act. The star product of any two smooth functions *f* and *g* defined over the phase space can be expressed in the differential form by the shift formula

4 $$\begin{aligned} (f*g)(x,p) = f\left( x+\frac{i\hbar }{2}\overrightarrow{\partial _p}, p-\frac{i\hbar }{2}\overrightarrow{\partial _x} \right) g(x,p). \end{aligned}$$

Besides the Weyl symbols of dynamical variables, a description of the quantum system in phase space also requires the concept of the states of the system. Therefore the states are represented by joint distributions of canonically conjugate variables (position and momentum), known as quasi-distribution functions^32–34^. One of them is the WDF, which is defined as the Weyl transform of the density operator ${\hat{\rho }}(t)$. The general form of the WDF is given by^31^

5 $$\begin{aligned} \rho (x,p; t) = \frac{1}{2\pi \hbar } \int dX \left\langle x+\frac{X}{2}\left| {\hat{\rho }}(t) \right| x-\frac{X}{2} \right\rangle \exp \left( -\frac{ipX}{\hbar }\right) . \end{aligned}$$

The WDF is bounded^21^ by the relation $|\rho (x,p; t)| \le 1/\pi \hbar$, and satisfies the normalization condition in the form

6 $$\begin{aligned} \int {dxdp}\; \rho (x,p;t) =1. \end{aligned}$$

The side effect of the Weyl transform of the density operator is that the WDF can take negative values in some regions of the phase space. This negativity of the WDF indicates the quantumness of the state, as was mentioned in Introduction.

Additional properties which make the WDF a convenient tool for analysis of the states in phase space are its moments. Especially, the zeroth moments of the WDF give correct marginal distributions with respect to position,

7 $$\begin{aligned} n(x;t) = \int {dp}\; \rho (x,p;t), \end{aligned}$$

and momentum,

8 $$\begin{aligned} {\widetilde{n}}(p;t) = \int {dx}\rho (x,p;t), \end{aligned}$$

which are interpreted as the probability densities in real and momentum space, respectively. Although the WDF is only a quasi-probability distribution, it can be applied to calculate the expectation value of any dynamical variable in the same way as in statistical mechanics,

9 $$\begin{aligned} \langle \Omega (t)\rangle = \int {dxdp}\; \Omega _{W}(x,p)\rho (x,p;t). \end{aligned}$$

It means that the expectation value of the dynamical variable $\Omega (x, p)$ represented by the corresponding Weyl symbol in an admissible state of the system is equal to the average of the Weyl symbol $\Omega _W(x, p)$ weighted with the WDF on the phase space. The time dependence of the expectation value is a consequence of the time evolution of the WDF for which the equation of motion can be written in the Moyal form as

10 $$\begin{aligned} i\hbar \partial _t\rho (x,p;t) = \left\{ H_W(x,p),\rho (x,p;t)\right\} _{\star }, \end{aligned}$$

where the curly brackets $\left\{ \cdot , \cdot \right\} _{\star }$ denote the skew-symmetric part of the star-product, $\left\{ f,g\right\} _{\star }(x,p):=(f*g)(x,p)-(g*f)(x,p)$, which is referred to as the Moyal bracket. In general, the Moyal bracket is a power series in $\hbar$,

11 $$\begin{aligned} \left\{ f,g\right\} _{\star }(x,p) = \left\{ f,g\right\} (x,p) + O(\hbar ^2), \end{aligned}$$

where the bracket $\left\{ \cdot,\cdot\right\}$ stands for the Poisson bracket. Therefore in the classical limit, as $\hbar \rightarrow 0$, the Moyal bracket reduces to the Poisson bracket

12 $$\begin{aligned} \lim _{\hbar \rightarrow 0}\left\{ f,g\right\} _{\star }(x,p) = \left\{ f,g\right\} (x,p). \end{aligned}$$

Application of the shift formula to the RHS of Eq. (10) allows one to write the equation of motion in the form

13 $$\begin{aligned} i\hbar \partial _t\rho (x,p;t) = \left[ H_W\left( x+\frac{i\hbar }{2}\partial _p, p-\frac{i\hbar }{2} \partial _x\right) \right. - H_W \left. \left( x-\frac{i\hbar }{2}\partial _p,p+\frac{i\hbar }{2}\partial _x \right) \right] \rho (x,p;t), \end{aligned}$$

though after some algebraic manipulation it can be simplified to

14 $$\begin{aligned} i\hbar \partial _t \rho (x,p;t) = -i\hbar \frac{p}{m}\partial _x\rho (x,p;t) + \bigg [ U\left( x+\frac{i\hbar }{2}\partial _p\right) - U\left( x-\frac{i\hbar }{2}\partial _p\right) \bigg ] \rho (x,p; t), \end{aligned}$$

which is more transparent for physical interpretation. Namely, if we expand the potential energy terms $U(x\pm (i\hbar /2) \partial _p)$ into power series about the point *x*, then we can transform Eq. (14) to the form of the deformed Liouville equation,

15 $$\begin{aligned} \partial _t \rho (x,p;t) + \frac{p}{m}\partial _x\rho (x,p;t) -\left[ \partial _xU(x)\right] \partial _p\rho (x,p;t) = \sum _{r=1}^{\infty } \frac{1}{(2r+1)!} \left( \frac{\hbar }{2i}\right) ^{2r} \partial _x^{2r+1}U(x) \partial _p^{2r+1}\rho (x,p;t). \end{aligned}$$

Some important observations can be made about this equation. First of all, the RHS explicitly depends on the Planck constant and its even powers. In the classical limit, $\hbar \rightarrow 0$, this term vanishes and Eq. (15) reduces to the classical Liouville equation. In this case the WDF evolves in time according to the classical equation of motion, and quantumness is included only in the prepared state. On the other hand, in the quantum limit ($\hbar \ne 0$) the RHS of Eq. (15) represents quantum corrections to the Liouville equation. Hence, the quantum dynamics is often regarded as a deformation of classical dynamics in phase space^52,53^. In this approach, the quantity $i\hbar /2$ is called the deformation parameter. Its meaning for the theory is twofold: firstly, the parameter enables one to proceed to the non-commutative regime of the phase space; secondly, it makes the dynamics of the WDF fully quantum with all of its attendant consequences. Finally, we note that the RHS of Eq. (15) vanishes if the potential is given by a polynomial of at most quadratic order. This has significant implications for the mathematical description of the WDF dynamics, because its quantum equation of motion is identical to the classical Liouville equation.

### Computational method

The solution of the Moyal equation allows one to determine the time evolution of the WDF provided that the initial condition is known. On the other hand, because of the mathematical complexity of this equation analytical solutions can be found in just a few cases. Therefore, in many physically interesting situations a numerical approach is needed to solve the equation. Among the existing numerical schemes developed for this type of equation^54–59^, the spectral split-operator method^60^ seems to be highly efficient^61–65^. This method allows us to look at the Moyal equation (14) as an example of a continuous dynamical system in phase space for which there exists a unitary time evolution operator $\mathscr {{\hat{U}}}(t_i-t_0)$ such that

16 $$\begin{aligned} \rho (x,p;t_i) = \mathscr {{\hat{U}}}(t_i-t_0)\rho (x,p; t_0), \end{aligned}$$

where $\rho (x,p;t_i)$ is the WDF at an arbitrary time instant $t_i$, and $\rho (x,p;t_0)$ corresponds to the WDF defined at the initial time $t_0$. In turn, the explicit form of the time evolution operator is given by the following formula

17 $$\begin{aligned} \mathscr {{\hat{U}}}(t_i-t_0) = \exp \left[ -\frac{i}{\hbar }\left( {\hat{T}}+{\hat{U}}\right) (t_i-t_0) \right] . \end{aligned}$$

In this notation, the operators ${\hat{T}}=-i\hbar (p/m)\partial _x$ and ${\hat{U}}=U(x+(i\hbar /2)\partial _p)-U(x-(i\hbar /2)\partial _p)$ represent the kinetic and potential parts of the Moyal equation (14) respectively. In computer simulations we obtain the time evolution of the WDF by applying the operator $\mathscr {{\hat{U}}}(\Delta t)$ repeatedly on the WDF, where $\Delta t$ is the time increment corresponding to a single step of the computations. The main idea of the application of the spectral split-operator method to the Moyal equation is based on the observation that the operators ${\hat{T}}$ and ${\hat{U}}$ do not commute. Hence the operator expression $\exp [-(i/\hbar )({\hat{T}}+{\hat{U}})\Delta t]$ acting on the WDF is much harder to compute than the results of separate operators $\exp [-(i/\hbar ){\hat{T}}\Delta t]$ and $\exp [-(i/\hbar ){\hat{U}}\Delta t]$ acting on the same function. To bypass this difficulty, the symmetric Strang splitting formula is applied^65–67^, i.e.

18 $$\begin{aligned} \exp \left[ -\frac{i}{\hbar }\left( {\hat{T}}+{\hat{U}}\right) \Delta t\right] = \exp \left( -\frac{i}{2\hbar }{\hat{T}}\Delta t\right) \exp \left( -\frac{i}{\hbar }{\hat{U}}\Delta t\right) \exp \left( -\frac{i}{2\hbar }{\hat{T}}\Delta t\right) + O\left( \Delta t^3\right) . \end{aligned}$$

Although this basic splitting formula is sufficient for our needs, let us note that the high-order variants of the splitting formula are more precise, but also unavoidably more complicated when applied to the Moyal equation, as has been discussed in Ref.^68^. Going back to the symmetric Strang splitting formula (18), it can be noted that each operator is unitarily equivalent to some multiplication operator owing to the adequate Fourier transform, specifically

19 $$\begin{aligned} \exp \left( -\frac{i}{2\hbar }{\hat{T}}\Delta t\right) = {\mathscr {F}}^{\lambda \rightarrow x} \exp \left( -\frac{i}{2m}p\lambda \Delta t\right) {\mathscr {F}}_{x\rightarrow \lambda } \end{aligned}$$

and

20 $$\begin{aligned} \exp \left( -\frac{i}{\hbar }{\hat{U}}\Delta t\right) = {\mathscr {F}}^{\theta \rightarrow p} \exp \bigg \{-\frac{i}{\hbar } \bigg [U\left( x-\frac{\theta }{2}\right) - U\left( x+\frac{\theta }{2}\right) \bigg ]\Delta t\bigg \} {\mathscr {F}}_{p\rightarrow \theta }, \end{aligned}$$

where the symbol ${\mathscr {F}}_{x\rightarrow \lambda }$ denotes the ordinary Fourier transform for the *x* variable with dual variable $\lambda$. In turn, the symbol ${\mathscr {F}}^{\lambda \rightarrow x}$ is the inverse Fourier transform for the variable $\lambda$ with dual variable *x*. A similar notion applies to variables $\theta$ and *p*. In both cases the symmetric convention of the Fourier transform is applied. Combining Eqs. (16) and (17), and taking into account the expressions given by Eqs. (19) and (20), we obtain the formula for a single step of the time evolution of the WDF in the form

21 $$\begin{aligned} \rho (x,p;t_0+\Delta t)\approx & {} {\mathscr {F}}^{\lambda \rightarrow x} \exp \left( -\frac{i}{2m}p\lambda \Delta t\right) {\mathscr {F}}_{x\rightarrow \lambda }^{\theta \rightarrow p} \exp \left\{ - \frac{i}{\hbar } \left[ U\left( x-\frac{\theta }{2}\right) - U\left( x+\frac{\theta }{2}\right) \right] \Delta t \right\} \nonumber \\&\times {\mathscr {F}}_{p\rightarrow \theta }^{\lambda \rightarrow x} \exp \left( -\frac{i}{2m}p\lambda \Delta t\right) {\mathscr {F}}_{x\rightarrow \lambda } \rho (x,p;t_0). \end{aligned}$$

In order to perform numerical simulations of the time evolution of the WDF based on Eq. (21) we limit the phase space ${\mathbb {R}}\times {\mathbb {R}}$ to the computational box of size $[-L_x,L_x]\times [-L_p,L_p]$ and discretize the phase space variables as

22 $$\begin{aligned} x_m=-L_x+m\Delta x,\quad p_n=-L_p+n\Delta p, \end{aligned}$$

where $\Delta x=2L_x/N_x$, $\Delta p=2L_p/N_p$, and the size of the computational grid is $N_x\times N_p$. In our calculations, the Fourier transforms are approximated by the discrete Fourier transforms, efficiently computed using the fast Fourier transform (FFT) algorithm. Because of the properties of the discrete Fourier transform, the dual variables have to be shifted and therefore we use the following discretization,

23 $$\begin{aligned} \lambda _k=\left\{ \begin{array}{ll} -L_\lambda +\left( k+\frac{N_x}{2}\right) \Delta \lambda , &{} k=0,1,...,\frac{N_x}{2} -1\\ -L_\lambda +\left( k-\frac{N_x}{2}\right) \Delta \lambda , &{} k=\frac{N_x}{2},...,N_x-1 \end{array} \right. \end{aligned}$$

and

24 $$\begin{aligned} \theta _l=\left\{ \begin{array}{ll} -L_\theta +\left( l+\frac{N_p}{2}\right) \Delta \theta , &{} l=0,1,...,\frac{N_p}{2} -1\\ -L_\theta +\left( l-\frac{N_p}{2}\right) \Delta \theta , &{} l=\frac{N_p}{2},...,N_p-1 , \end{array} \right. \end{aligned}$$

where $$\Delta \lambda =\pi /L_x$$, $$\Delta \theta =\pi /L_p$$, $$L_\lambda =N_x\Delta \lambda /2$$, and $$L_\theta =N_p\Delta \theta /2$$.

The numerical calculations were performed in atomic units (a.u.), i.e. $\hbar =e=m=1$. For these calculations we adopted the following parameters of the computational grid: $N_x=N_p=1024$ as mesh points for the coordinate *x* and momentum *p*, respectively, with $L_x=1500$ a.u., $L_p=0.5$ a.u., and the time step $\Delta t=10$ a.u.

### Preparation of the initial state and its properties

According to Eq. (16), to proceed with the time evolution of the WDF we need to establish the initial condition. Because the WDF is the phase-space representation of the density operator we therefore construct the initial condition for the Moyal equation starting from the density operator, ${\hat{\rho }}(t)=\left| \psi (t)\rangle \langle \psi (t)\right|$, for the state given by Eq. (1). As a result we obtain the following expression

25 $$\begin{aligned} {\hat{\rho }}(t) = A^2 \bigg \{ \left( 1-\beta \right) \left| \phi _1(t)\rangle \langle \phi _{1}(t)\right| + \beta \left| \phi _{2}(t)\rangle \langle \phi _{2}(t)\right| + \sqrt{\beta \left( 1-\beta \right) } \bigg ( e^{i\vartheta } \left| \phi _{2}(t)\rangle \langle \phi _{1}(t)\right| + e^{-i\vartheta } \left| \phi _{1}(t)\rangle \langle \phi _{2}(t)\right| \bigg ) \bigg \}. \end{aligned}$$

It is widely known that the last term in Eq. (25), related to quantum interference between states $|\phi _{1}(t)\rangle$ and $|\phi _{2}(t)\rangle$, is easily affected by decoherence processes and is most often destroyed by them^7^. This observation allows us to adopt a certain strategy, namely to parameterize the interference term using a scaling parameter $\Gamma$ taken in the range $\left[ 0, 1\right]$. As a result, the density operator can be written as^69,70^

26 $$\begin{aligned} {\hat{\rho }}(t) = A^2_{\Gamma }\bigg \{ (1-\beta )\left| \phi _{1}(t)\rangle \langle \phi _{1}(t)\right| + \beta \left| \phi _{2}(t)\rangle \langle \phi _{2}(t)\right| + \Gamma \sqrt{\beta (1-\beta )}\left( e^{i\vartheta } \left| \phi _{2}(t)\rangle \langle \phi _{1}(t)\right| + e^{-i\vartheta }\left| \phi _{1}(t)\rangle \langle \phi _{2}(t)\right| \right) \bigg \}. \end{aligned}$$

When $\Gamma =0$, it corresponds to a statistical mixture of these two states, whereas in the case $\Gamma =1$, it corresponds to the coherent superposition of the states.

Before we proceed, let us make some remarks about the efficiency of numerical algorithm based on the Schrödinger representation, which could be used as an alternative way of solving this problem. We start from the observation that diagonalization of the density operator in the form given by Eq. (25) leads to the following expression

27 $$\begin{aligned} {\hat{\rho }}(t) \propto (1+\Gamma )\left| \phi _{even}(t)\rangle \langle \phi _{even}(t)\right| + (1-\Gamma )\left| \phi _{odd}(t)\rangle \langle \phi _{odd}(t)\right| , \end{aligned}$$

where $|\phi _{even{/}odd}(t)\rangle =\sqrt{1-\beta }|\phi _{1}(t)\rangle \pm \sqrt{\beta } \exp {(i\vartheta )}|\phi _{2}(t)\rangle$ can be called the even and odd Schrödinger cat states, respectively. In this case, the time-evolution of the diagonal form of the density matrix can be obtained by solving the Schrödinger equation for the even and odd states, separately. A single time step of such evolution has the complexity $O(N_x\ln N_x)$ when the spectral split-operator method is used^71^. It consists of element-wise one-dimensional array multiplications and the one-dimensional FFTs which have complexity $O(N_x)$ and $O(N_x\ln N_x)$, respectively. Such approach is therefore an effective method if we are interested only in characteristics which can be straightforwardly calculated from the wave functions in the position or momentum representation. Let us also note that the transformation between these representations can be computed with the one-dimensional FFT with computational complexity $O(N_x\ln N_x)$. At first glance, this approach seems to be quite effective method of the solution of the dynamical problem. However, the obtained results do not allow direct investigation of the quantumness of the state which is one of the central issues of our research. It requires calculating the WDF (5) which can be expressed as $\rho (x,p)=\rho _{r}(\sqrt{2}x,\sqrt{2}p; t)$, where

28 $$\begin{aligned} \rho _{r}(x,p; t) = \frac{1}{\sqrt{2}\pi \hbar } \int dX \left\langle \frac{x+X}{\sqrt{2}}\left| {\hat{\rho }}(t) \right| \frac{x-X}{\sqrt{2}}\right\rangle \exp \left( -\frac{ipX}{\hbar }\right) , \end{aligned}$$

so it can be obtained in each time step from the density matrix $\langle x|{\hat{\rho }}(t) |x' \rangle$ by three succeeding transformations, namely rotation by the angle $\pi /4$, the partial Fourier transform in the second variable, and rescaling. The high quality rotation of 2D discrete grid can be calculated by FFT-based algorithms^72,73^ with the computational complexity $O(N_x^2\ln N_x)$. Simpler and less computationally demanding algorithms can be used like the nearest neighbour method, but it would lead to distortions especially in fine structures of the density matrix. Anyways, the next step of computing WDF is the partial Fourier transform in the second variable which can be computed using FFT with complexity $O(N_x^2\ln N_x)$, and rescaling can be achieved by simply reinterpreting spacing between the grid points. On the other hand, a single time step of our method (21) has the computational complexity $O(N_x^2\ln N_x)$ as $N_p=O(N_x)$ (in many applications $N_p=N_x$ or $N_p=N_x/2$) and the algorithm is composed of element-wise array multiplications and the two-dimensional partial FFTs which have complexities $O(N_x^2)$ and $O(N_x^2\ln N_x)$, respectively. Hence, we can conclude that the complexities of both methods are similar when dynamical characteristics based directly on the knowledge of the WDF are required, as in the case of the non-classicality parameter^36^. However, the advantage of the phase-space approach is revealed when we consider the initial states which cannot be decomposed as a mixture of a small number of pure states, like e.g. the Gibbs thermal states of the harmonic oscillator.

Now, let us get back to the discussion on the modification of the considered state given by Eq. (26). Its parameterization with $\Gamma$ allows us to influence the interference term. It is quite important because if we assume that the initial state of the system is represented by the density operator in that form, then we can interpret the parameter $\Gamma$ as the factor responsible for the quality of the prepared quantum state. Hence, $\Gamma$ is called the quality parameter of the state. In Ref.^69^, authors proposed a simple decoherence model for superposition of coherent states that produces states like in Eq. (26). Eq. (25) allows one to determine the WDF for the superposition of two distinguishable quantum states. To achieve that, time-independent wave functions $\phi _{1}$ and $\phi _{2}$ are used. Both have a localized form in the position representation as Gaussian wave packets centered around the points $x_1$ and $x_2$, respectively. Assuming that the initial widths are equal to $\delta _x$ and the average momentum is $p_0$ we can write (for $k=1,2$)

29 $$\begin{aligned} \langle x|\phi _{k}\rangle = \root 4 \of {\frac{1}{2\pi \delta _x^2}} \exp \left[ -\frac{\left( x-x_{k}\right) ^2}{4\delta _x^2} + \frac{i}{\hbar } p_0 x\right] , \end{aligned}$$

which minimizes the Heisenberg uncertainty principle $\delta _x^2\delta _p^2=(\hbar /2)^2$. Substituting Eq. (26) into Eq. (5) and taking into account Eq. (29) produces the WDF which amounts to two Gaussians and an oscillating term with the Gaussian envelope between them, that is

30 $$\begin{aligned} \rho (x,p)= & {} A_\Gamma ^2 \frac{\left( 1-\beta \right) }{\pi \hbar } \exp \left[ -\frac{\left( x-x_1\right) ^2}{2\delta _x^2} - \frac{2\delta _x^2\left( p-p_0\right) ^2}{\hbar ^2}\right] + A_\Gamma ^2 \frac{\beta }{\pi \hbar } \exp \left[ -\frac{\left( x-x_2\right) ^2}{2\delta _x^2} - \frac{2\delta _x^2\left( p-p_0\right) ^2}{\hbar ^2}\right] \nonumber \\&+ 2A_\Gamma ^2\Gamma \frac{\sqrt{\beta \left( 1-\beta \right) }}{\pi \hbar } \cos \left[ \vartheta + \frac{p-p_0}{\hbar } \left( x_1 - x_2 \right) \right] \exp \left[ -\frac{\left( x-\frac{ x_1 + x_2 }{2}\right) ^2}{2\delta _x^2}-\frac{2\delta _x^2\left( p-p_0\right) ^2}{\hbar ^2} \right] . \end{aligned}$$

In the formula above, $x_1-x_2=d$ represents the distance between the centers of mass of the Gaussians if $x_1 > x_2$, while the normalization factor $A_{\Gamma }$ equals

31 $$\begin{aligned} A_\Gamma= & {} \left[ 1+2\Gamma \sqrt{\beta \left( 1-\beta \right) } \exp \left( -\frac{d^2}{8\delta _x^2} \right) \cos \vartheta \right] ^{-\frac{1}{2}}. \end{aligned}$$

**Figure 1:**
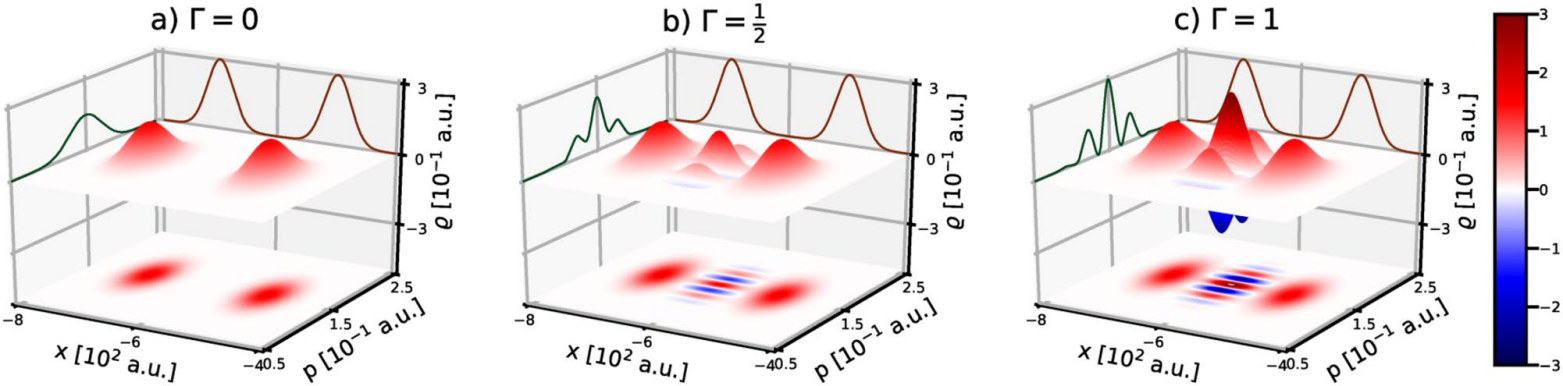
Influence of the quality parameter $\Gamma$ on the Wigner form of the initial DSC-state with $\vartheta =0$. Marginal distributions (not to scale with WDF units) with respect to position and momentum are shown on the sidewalls with brown and green solid lines respectively.

**Figure 2:**
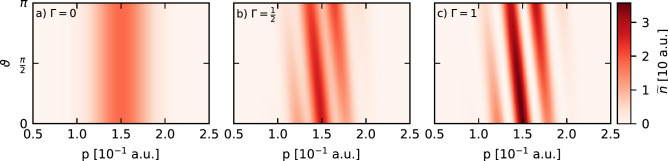
Influence of the relative phase $\vartheta$ on the probability density in momentum space, ${\widetilde{n}}(p)$, for fixed values of the quality parameter $\Gamma$. For **a** $\Gamma =0$, and ${\widetilde{n}}(p)$ does not depend on $\vartheta$.

We refer to the WDF given by Eq. (30) as the phase-space representation of the defective Schrödinger cat state (DSC-state). This state depends on five parameters: the distance *d*, the initial width $\delta _x$, the relative phase $\vartheta$, the quality factor $\Gamma$, and $\beta$ which controls the amplitude ratio. For the further analysis we assume that the amplitudes of the Gaussians, their initial widths, as well as the distance between the Gaussians are fixed, with $\beta =0.5$, $\delta ^2_x=500$ a.u. and $d=200$ a.u. This choice of the parameters guarantees that the considered DSC-state corresponds to the superposition of two distinguishable localized Gaussians in real space. Additionally, we assume that the initial average momentum of each of the DSC-states equals $p_0=0.15$ a.u. The influence of various values of the $\Gamma$ and $\vartheta$ parameters on the DSC-state is presented in Figs. 1 and 2.

The quality parameter $\Gamma$, which influences the DSC-states as shown in Fig. 1, enters into the formula (30) in two ways: through the normalization factor $A^2_{\Gamma }$ and through the product $A^2_{\Gamma }\Gamma$. When $\Gamma$ changes from 1 to 0 (and when $\vartheta$ remains constant), there is no significant change in the normalization factor $A^2_{\Gamma }$, while the product $A^2_{\Gamma }\Gamma$ approaches zero almost linearly. The consequences are different for different terms in Eq. (30), namely the Gaussians almost do not feel these changes, but the cross term related to the quantum interference processes is effectively destroyed. Hence we observe different intensities of the interference fringes for the DSC-state depending on the parameter $\Gamma$, which is particularly evident in the limiting cases when the parameter $\Gamma$ is 0 or 1 (cf. Figs 1a,c). It shows that $\Gamma$ evidently regulates the strength of the interference fringes and therefore it can be used as a measure of the imperfection of the DSC-state.

The last parameter of the DSC-state to be considered is the relative phase $\vartheta$. It has a notable influence only on the interference term of the DSC-state, which is a consequence of the cosine function in Eq. (30). This influence is evident in the probability density in momentum space,

32 $$\begin{aligned} {\widetilde{n}}\left( p ; p_0 \right) = \frac{2\delta _x^2 A_\Gamma ^2}{\pi \hbar } \bigg \{ 1+2\sqrt{\beta (1-\beta )}\Gamma \cos \left[ \vartheta +\frac{\left( p-p_0\right) }{\hbar } d \right] \bigg \} \exp \left[ -\frac{2\delta _x^2\left( p-p_0\right) ^2}{\hbar ^2} \right] . \end{aligned}$$

Figure 2 displays this marginal distribution in momentum space for the relative phase $0 \le \vartheta \le \pi$ and the quality parameter $\Gamma \in \{0, 1/2, 1\}$. The distance *d* between the Gaussians used to prepare the DSC-state affects the number of maxima of the probability density ${\widetilde{n}}(p; p_0)$ visible in Fig. 2a,b, whereas the positions of those maxima are given by the formula $\vartheta +(1/\hbar )(p-p_0)d=\pm 2\pi n$ (where *n* is either a natural number or zero) and their heights (together with the amplitude of the probability density changes) are increased with larger values of $\Gamma$. Additionally, if $\vartheta$ is not an integer multiple of $\pi$, the probability density ${\widetilde{n}}(p; p_0)$ is not symmetric with respect to the average initial momentum $p_0$.

It should also be mentioned that the relative phase $\vartheta$, as well as the quality parameter $\Gamma$, have no significant influence on the probability density in real space, *n*(*x*), given by

33 $$\begin{aligned} n(x)= & {} \frac{A_\Gamma ^2}{\sqrt{2\pi \delta _x^2}} \Bigg \{ (1-\beta ) \exp \left[ -\frac{\left( x-x_1\right) ^2}{2 \delta _x^2}\right] + \beta \exp \left[ -\frac{\left( x-x_2\right) ^2}{2 \delta _x^2}\right] + 2\Gamma \sqrt{\beta (1-\beta )} \cos \vartheta \nonumber \\&\times \exp \left( -\frac{d^2}{8\delta _x^2}\right) \exp \left[ - \frac{\left( x-\frac{x_1+x_2}{2}\right) ^2}{2\delta _x^2} \right] \Bigg \}, \end{aligned}$$

since for the used simulation parameters the numerical value of the prefactor $\exp (-d^2/8\delta _x^2)$ is very small, approximately $0.45\times 10^{-5}$. Finally, it is worth noting that for the assumed average initial momentum $p_0$, the expectation value of the kinetic energy $\left\langle E_k\right\rangle _{DSC}\cong 0.0115$ a.u. is almost independent of the relative phase $\vartheta$ and the quality parameter $\Gamma$.

### Characterization of the states

Due to the non-classicality of the state in question, we determine the non-classicality parameter, $\delta$, which is defined as^36^

34 $$\begin{aligned} \delta = \int {dx dp}\; \left[ \left| \rho (x,p)\right| -\rho (x,p) \right] . \end{aligned}$$

According to this definition, a non-zero value of the parameter indicates the existence of non-classical properties of the state. We note that the parameter $\delta (t)$ can also be expressed as a sum of the moduli of integrals over those regions of the phase space where the WDF is positive and negative^74^. As a result, we can conclude that the non-classicality parameter equals double the area occupied by the negative part of the WDF^36^.

Another basic quantity which characterizes the DSC-state is its time-dependent extent in phase space, which can be regarded as a degree of localization in that space. This property can be extracted from calculations of the differential Shannon entropy which is defined in general for any probability density distribution, *P*(*r*), of a dimensional continuous random variable, *r*, as follows^75^:

35 $$\begin{aligned} S^{(r)} = - \int {dr}\; P(r)\ln {\left[ P(r) u_r\right] }. \end{aligned}$$

where $u_r$ is a fixed unit of the *r*-variable^29^. In our case, the variable *r* may represent the position *x* or the wave vector (rescaled momentum) $p\rightarrow p/\hbar =k$, with the corresponding probability distribution given by *n*(*x*; *t*) or ${\widetilde{n}}(k;t)$. In this way the differential entropies $S^{(x)}(t)$ and $S^{(k)}(t)$ can be regarded as dynamical measures of the degree of state localization in position and momentum space, respectively. The significance of these entropic measures for bi- or multi-modal distributions is explained by suggestive examples in Ref.^29^. In accordance with the argumentation presented there, we conclude that they are more than adequate to quantify the localization of multi-modal distributions than the more widely used standard deviation.

To perform the analysis of the degree of localization of the DSC-state in both phase-space variables, we can use the entropic uncertainty principle expressed by the formula^29.^

36 $$\begin{aligned} S^{(x)}(t) + S^{(k)}(t) = - \int {dx}\; n(x;t)\ln {n(x;t)} - \int {dk}\; {\widetilde{n}}(k;t)\ln {{\widetilde{n}}(k;t)} \ge \ln (e \pi ). \end{aligned}$$

This expression is independent of the choice of the units since $u_x=1/u_k$ holds for both pure and mixed states and is saturated by coherent Gaussian states, as in the case of the Heisenberg uncertainty relation. In fact, the entropic uncertainty relation is stronger, namely it satisfies the following double inequality^76^

37 $$\begin{aligned} \sigma _x(t)\sigma _k (t) \ge \frac{1}{2e\pi }\exp \left[ S^{(x)}(t)+S^{(k)}(t)\right] \ge \frac{1}{2}. \end{aligned}$$

## Results and discussion

The DSC-state discussed above [cf. Eq. (30)] is the initial condition for the Moyal equation of motion (14). It is solved numerically by applying the second-order split-operator method according to Eq. (18). By using this method we can investigate the dynamical properties of the considered state in a dispersive medium with an internal perturbation which breaks its homogeneity. This perturbation is taken in the form of a single obstacle which can mimic a structural defect or a dopant in a nanowire^77^. We model this obstacle as a barrier in the form of the repulsive Gaussian potential,

38 $$\begin{aligned} U(x) = U_0\exp \left[ -\frac{(x-X_B)^2}{2w^2}\right] , \end{aligned}$$

where $U_0$ is the strength of the potential located at the position $X_B$ and *w* determines the width of the barrier. We assume that the barrier is located in the middle of the simulation region, i.e., $X_B=-200$ a.u, and the other two parameters of the barrier are $U_0=0.008$ a.u. and $w^2=50$ a.u.

The asymptotical form of the probability density distribution in the momentum space after interacting with the barrier is given by

39 $$\begin{aligned} {\widetilde{n}}(p;p_0) = {\widetilde{n}}_{refl}(p;p_0) +{\widetilde{n}}_{trans}(p;p_0), \end{aligned}$$

where the first (second) part on the RHS corresponds to the reflected (transmited) part of the wave packet. Both of them can be expressed via the initial probability density distribution in the momentum space ${\widetilde{n}}(p;p_0)$ before interaction with the barrier and either reflection or transmission coefficient as was presented in^78^

40 $$\begin{aligned} {\widetilde{n}}_{refl}(-p;p_0) = {\widetilde{n}}(p;p_0)R(p), \end{aligned}$$

and

41 $$\begin{aligned} {\widetilde{n}}_{trans}(p;p_0) = {\widetilde{n}}(p;p_0)T(p), \end{aligned}$$

where the minus sign in ${\widetilde{n}}_{refl}(-p;p_0)$ is caused by the fact that this part of the wave packet changed direction of movement after interaction with barrier. Interaction with the potential barrier leads to the possibility of two non-classical effects. First, a particle approaching the barrier but with an energy below the top of the barrier may tunnel through it, which is called the below-barrier penetration (BBP). The momentum corresponding to the energy equal to the barrier height $U_0$ is $p_B=\sqrt{2mU_0}$ and the probability of BBP is given by

42 $$\begin{aligned} P_{BBP}(p_0) = \int _0^{p_B} dp \, {\widetilde{n}}_{trans}(p;p_0) = \int _0^{p_B} dp \, T(p){\widetilde{n}}(p;p_0). \end{aligned}$$

The second non-classical effect is that a particle approaching the barrier with an energy above the top of the barrier may be reflected, which is called the above-barrier reflection (ABR) and generates backscattering diffraction^17^.

The theoretical explanation of this classically forbidden reflection is based on the methods combining the perturbative and the WKB-semiclassical approches^79^. These results lead to the conclusion that the effective potential for the WKB wave function differs from the modeled potential, and the difference between them is responsible for the considered quantum reflection. The phase-space picture for this problem^79,80^ shows that the above-barrier reflection is indicated by a virtual trajectory parallel to the momentum axis connecting the phase-space trajectories corresponding to the opposite momenta. This effect is interpreted as the tunneling in the momentum space^81^.

For the DSC-state, analogously to BBP, the probability of ABR is calculated as

43 $$\begin{aligned} P_{ABR}(p_0) = \int _{p_B}^{\infty } dp \, {\widetilde{n}}_{refl}(-p;p_0) = \int _{p_B}^{\infty } dp \, R(p){\widetilde{n}}(p;p_0). \end{aligned}$$

**Figure 3:**
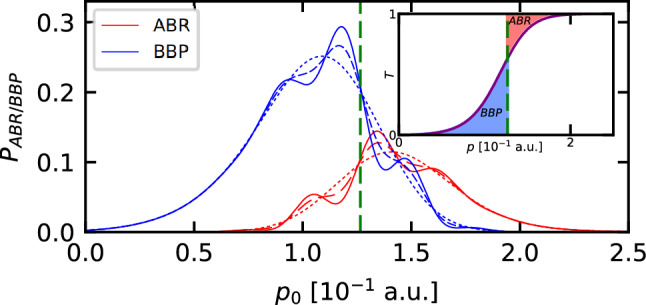
Probabilities of BBP and ABR for the initial DSC-state interacting with the Gaussian barrier. Solid, dashed, and dotted lines correspond accordingly to $\Gamma =1$, $\Gamma =0.5$ and $\Gamma =0$; the vertical green dashed line corresponds to the momentum $p_B$. The inset shows the transmission coefficient *T*(*p*).

Probabilities of BBP and ABR for the considered Gaussian barrier and the DSC-state are presented in Fig. 3 as functions of the average momentum $p_0$, with the vertical green dashed line corresponding to the momentum $p_B$.

We can see that the BBP effect is most significant for $p_0$ slightly below $p_B$, while the ABR effect is at its most significant for $p_0$ slightly above $p_B$. Since we are interested in the latter regime, it justifies our choice of the average momentum $p_0=0.15$ a.u. At this point let us note that the case under consideration corresponds to the situation in which the expectation value of the kinetic energy of the initial DSC-state is greater than the maximum of the potential energy $U_0$. The proportion of this state which can be found above the barrier is calculated as follows,

44 $$\begin{aligned} \int _{p_B}^{\infty } dp \, {\widetilde{n}}(p;p_0) \approx 0.8, \end{aligned}$$

which means that approximately $20\%$ of the state forms a low-energy tail that impinges on the barrier. As is shown in the inset of Fig. 3, the transmission coefficient *T*(*p*) calculated for the Gaussian barrier reveals no resonances as it is monotonically increasing. Besides, for wider Gaussian barriers the transmission coefficient is more and more steep (the red and blue areas on embedded plot get smaller and smaller) which results in BBP and ABR effects being greatly suppressed. The initial DSC-state is located to the left of the barrier at a distance greater than its initial spatial dimension. The state moves to the right with the same momentum as in the previously discussed case, and its other parameters are also unchanged.

**Figure 4:**
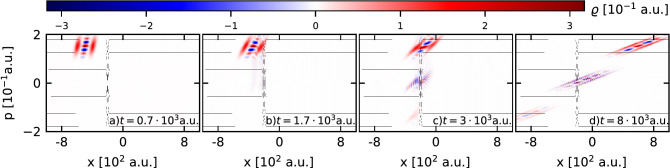
The phase-space snapshots of the DSC-state at different times during the interaction with the Gaussian potential barrier. The contour lines represent equipotential lines of the classical Hamiltonian for the considered system.

**Figure 5:**
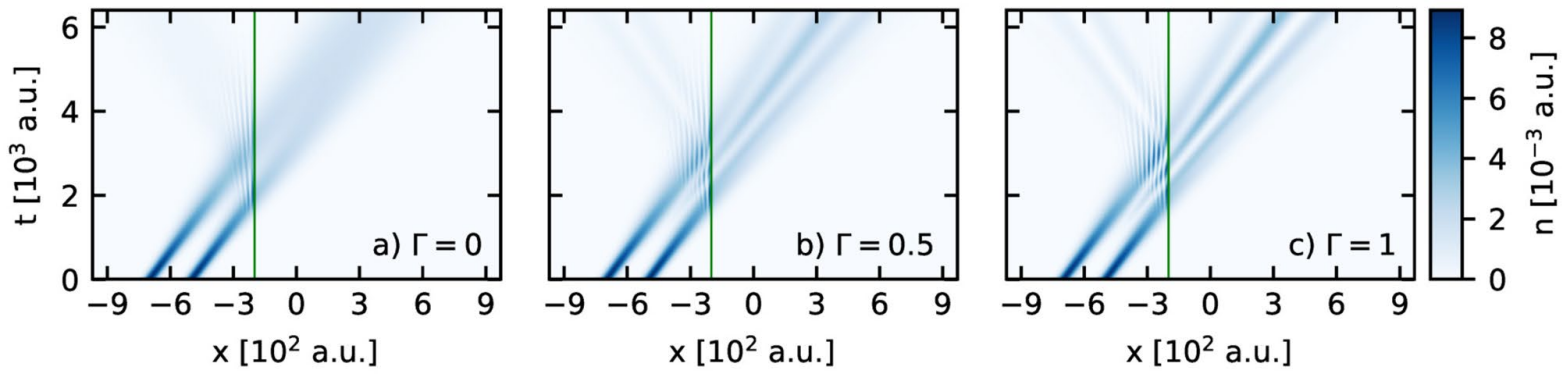
The spatio-temporal pattern of the probability density for the DSC-state in the presence of the potential barrier for different values of the quality parameter: (**a**) $\Gamma =0$, (**b**) $\Gamma =0.5$ and (**c**) $\Gamma =1$. The vertical solid line indicates the position of the potential barrier.

We performed a series of computational experiments based on the numerical solution of the Moyal equation in which the DSC-state interacts with the Gaussian barrier. As a result we obtained the phase-space picture of the DSC-state evolution during its interaction with the barrier for different values of the quality parameter $\Gamma$. Sample phase-space snapshots of the state with the parameter $\Gamma =1$ at characteristic time instants are shown in Fig. 4 (a movie is available in the Supplemental Material). Here we can observe the process of formation of the secondary interference term near the barrier. Moreover, for this set of the simulation parameters, the interaction of the incoming DSC-state with the barrier generates the reflected and outgoing parts independently of the parameter $\Gamma$. The dynamics of this process in real space are visualized in Fig. 5, where we present the spatio-temporal probability density for different values of the parameter $\Gamma$.

Based on these patterns, we can characterize the process as follows. At large distances from the potential barrier the initial DSC-state moves along two well-separated channels which expand over time due to the dispersion properties of the Gaussian packets that form the state. In this case the influence of the quality parameter $\Gamma$ on the dynamics of the DSC-state can be neglected, because it corresponds to the first moments of the free propagation. However, the significance of the quality parameter manifests itself near the barrier. Careful analysis of Fig. 5 allows the observation not only of interference fringes, but also a cusp in the density *n*(*x*; *t*) emerging between the channels due to the primary interference. Evidently, intensity and the place of formation of the cusp depend on the value of the quality parameter (cf.  Fig. 5b,c), and the process of passing through the barrier does not destroy it. In turn, this effect is not observed in Fig. 5a where the time evolution of the statistical mixture of two Gaussians is presented. The performed simulations suggest that the primary quantum interference which is a part of the DSC-state, is enhanced by the secondary interference which stems from the interaction of the state with the barrier. Moreover, the primary quantum interference contributes to more and more pronounced interference fringes in front of the barrier and therefore we observe more and more clearly visible oscillations of the density in this region. Visualizations and details of the time evolution of both marginals are available as movies featured in the Supplemental Material.

**Figure 6:**
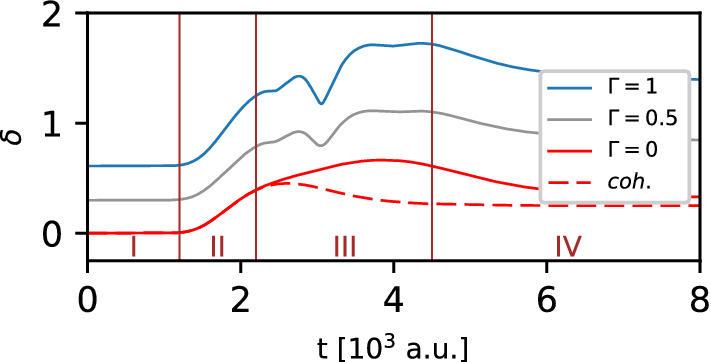
Influence of the quality parameter $\Gamma$ on the non-classicality parameter for the DSC-state with the relative phase $\vartheta =0$, which interacts with the Gaussian barrier. The dashed line corresponds to a single Gaussian wavepacket.

Proceeding as before, we begin our analysis of the dynamical properties of the DSC-state in the presence of the Gaussian barrier with the investigation of the time dependence of the non-classicality parameter $\delta =\delta (t)$. It allows us to quantify the significance of the secondary interference during the interaction of the state with the barrier. Figure 6 displays the non-classicality parameter as a function of time for several values of the quality parameter $\Gamma$, and a fixed value of the relative phase $\vartheta =0$.

The obtained results demonstrate that the non-classicality parameter increases in time. Moreover, this increase evidently depends on the preparation of the initial state through the parameter $\Gamma$. To analyze these changes it is convenient to divide the entire simulation time into four intervals. Interval I includes the time period from 0 to 1200 a.u. Therein the non-classicality parameter remains constant because the DSC-state does not feel the presence of the barrier, and therefore its time evolution corresponds to free propagation of the DSC-state. Interval II extends from 1200 to 2200 a.u. In this interval, the value of the non-classicality parameter gradually increases, which is caused by the interaction of the DSC-state with the barrier. As a result, the negative part of the corresponding WDF starts to increase and it makes additional contributions to the parameter $\delta$. This behavior should be understood as an announcement of the process of passing through the barrier. Interval III extends from 2200 to 4500 a.u. In this interval the non-classicality parameter shows some changes of the slope as one of the wave packets constituting the DSC-state is mainly behind the region of the potential barrier. Simultaneously, the reflected part emerges and starts to interact with the forthcoming second wave packet which is just starting to interact with the barrier. As a result, the quantum interference is enhanced or reduced depending on the relative phase. Finally, the interval IV extends from 4500 a.u. to the end of the simulation time. Therein the non-classicality parameter gradually decreases in time, and asymptotically approaches a constant value. In principle the process of passing the DSC-state through the barrier is completed.

**Figure 7:**
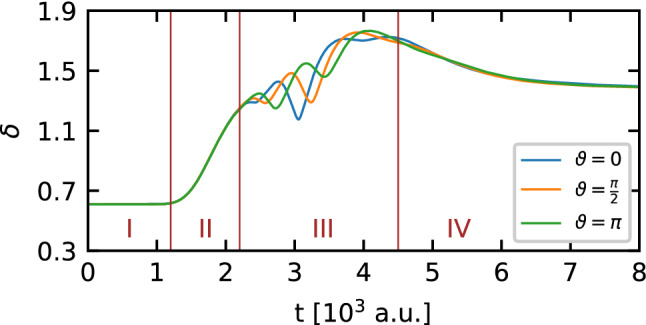
Influence of the relative phase $\vartheta$ on the non-classicality parameter for the DSC-state with the quality parameter $\Gamma =1$, which interacts with the Gaussian barrier.

**Figure 8:**
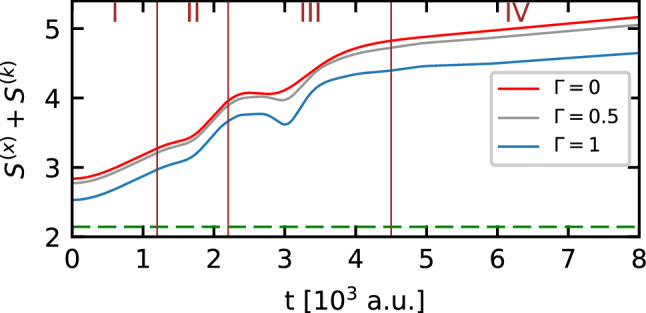
Effect of the barrier on the sum of the differential entropies of the DSC-state with the relative phase $\vartheta =0$ for different values of the quality parameter $\Gamma$. The dashed line corresponds to the mimimum value of the sum of differential entropies according to Eq. (36).

The observed changes of the non-classicality parameter in the intervals II and III prompted us to check the influence of the relative phase on this parameter, all the more because the influence of the relative phase is clearly visible in the probability density distribution of momentum of the initial DSC-state (cf. Fig. 2). In addition, we note that the momentum components of the DSC-state are redistributed during the interaction of the state with the barrier. The influence of the relative phase on the non-classicality parameter is shown in Fig. 7. For the analysis of the impact of the relative phase on the non-classicality parameter, the interval III is the most critical, since in this region quantum interference from two sources overlap: the primary interference presented in the initial DSC-state, and the secondary interference associated with the passage of the state through the potential barrier. Those two processes of interference can be amplified (constructive interference) or suppressed (destructive interference). Let us also note that this phenomenon changes in time, causing visible oscillations of the non-classicality parameter in the considered interval. By changing the relative phase of the initial state, which is encoded in the interference component of the DSC-state, we shift instants of time when the constructive and destructive interference dominate. This is the reason of the observed phase shift in the above-mentioned oscillations of the non-classicality parameter in the interval II of the DSC-state time evolution. Besides, we determine the extent of the DSC-state in the phase space by calculating the time-dependence of the sum of the differential entropies from Eq. (36). Results of the calculations are presented in Fig. 8. As before, the sum of the differential entropies for the considered state is sensitive to the quality parameter $\Gamma$, and reveals two points where we can observe a deviation from a simple monotonic behavior due to the perturbation of the homogeneity of the isolated system which influences the DSC-state: a kink in the interval II, and a minimum placed in interval III. Both these time intervals correspond to the interaction of the DSC-state with the potential barrier, and the changes in the shape of the function are closely related to the passage of subsequent packets forming the DSC-state through the barrier. Let us note that on arrival at the barrier, the first packet is compressed so that the entire DSC-state occupies a smaller area in space. Simultaneously, the quantum interference begins to appear, causing the DSC-state to become more extended in space. The same explanation can be used when the second packet passes the barrier, but there is an additional complication here due to the generation of the clearly visible reflected part of the DSC-state. Thus the area occupied by the state in the phase space is larger than at the beginning of the simulation.

## Concluding remarks

We have considered the dynamical properties of the family of non-classical states defined as the coherent superposition of two well separated Gaussian wavepackets, with a defect due to an imperfection in their preparation. This imperfection is described by a scaling parameter which controls the coherence of these states. The states created in this way are called defective Schrödinger cat states. The presented studies are based on the phase space formulation of quantum theory and the Wigner distribution function, and its equation of motion in the Moyal form. This approach is convenient for detecting non-classical effects which stem from the quantum corrections to the classical motion of the states of the isolated quantum system. It is also a well established method of research used for open quantum systems or hybrid systems, i.e., quantum-classical ones. Utilizing this approach we have shown that the quantumness of the considered states expressed by the negative part of the corresponding Wigner distribution function is sensitive to the scaling parameter. Quantitatively, this effect is examined by means of the time-dependent non-classicality parameter. Additionally, we have performed a complementary analysis investigating the localization degree of these non-classical states using the entropic measure expressed by the sum of differential entropies, which is a non symplectically-invariant measure of uncertainty in the phase space. As a direct consequence of the used measure, the degree of localization of the defective Schrödinger cat states changes with time. This analysis was dictated by the fact that the considered states resemble bi-modal distributions. The application of this measure allows us to show that the degree of localization also depends on the scaling parameter. This is a crucial result because an analysis based on the standard deviations of position and momentum misses this observation.

The presented results concern a dispersive medium isolated from the environment with the Gaussian barrier which breaks the homogeneity. The analysis is concentrated on the backscattering diffraction caused by reflection above the potential barrier. The setup of the above-barrier reflection regime has been motivated by the investigation of the scaling parameter’s influence on the backscattering diffraction of defective Schrödinger cat states. Our first observation is that this phenomenon occurs for the considered non-classical states independently of the value of the scaling parameter. This observation has measurable importance for quantum backscattering communication, namely the obtained results show that independently of the value of the scaling parameter, the backscattering diffraction always occurs for the defective Schrödinger cat states. Moreover, this phenomenon can be intensified by a proper choice of the parameters of the barrier and the incoming state, as it results from the expression for the probability of the above-barrier reflection. One interesting result which stems from the presented studies is that the reflected part has a form of nearly bi-modal distribution in the real space when the scaling parameter is different from zero. A careful analysis of this result allows us to state that both the reflected and the transmitted parts of the Wigner distribution function resemble the initial Wigner function of the defective Schrödinger cat state up to a shearing transformation. The emergence of the real-space multi-mode distribution in the reflected part of the considered state, as well as the single-mode distribution of this part, are shown in the Supplemental Material, which also includes the illustration of the Wigner function dynamics.

Apart from that, we have shown that the non-classicality parameter and the localization degree of the non-classical state in phase-space, increase in time with respect to their initial values. However these two quantities achieve a local minimum in a time interval which corresponds to the time during which the considered state remains in the immediate vicinity of the barrier. These minima are a consequence of partial compression of the state due to the presence of the barrier.

This setup of the computer simulations is closely related to detecting the quantum interference of the bimodal states in electronic systems. The experimental realization of this setup can be based on the system consisting of two quantum dots separated by a barrier^82^. However, such measurements seem to be extremely difficult^83,84^. Perhaps, the difficulties can be overcome with the protocol based on an electronic Mach–Zehnder interferometer coupled electrostatically to two quantum point contacts^85^.

Summarizing this discussion, we believe that the presented results have potential applications concerning the transfer of quantum states along dispersive media with defects and they may be inspiring for related experiments testing ideas of quantum communication. Moreover, these results allow for the development of the engineering of quantum backscattering communication, by controlling and steering of the reflected part of incoming non-classical states of light or matter, in the above-barrier reflection regime.

## Supplementary Information

Supplementary Movie 1.

Supplementary Movie 2.

## References

[1] Merzbacher E (1998). Quantum Mechanics.

[2] Geyer P (2016). Perspectives for quantum interference with biomolecules and biomolecular clusters. Phys. Scr..

[3] Bach R, Pope D, Liou S-H, Batelaan H (2013). Controlled double-slit electron diffraction. New J. Phys..

[4] Gerry CC, Knight PL (1997). Quantum superpositions and Schrödinger cat states in quantum optics. Am. J. Phys..

[5] Wineland DJ (2013). Nobel lecture: Superposition, entanglement, and raising Schrödinger’s cat. Rev. Mod. Phys..

[6] Weinbub J, Ferry DK (2018). Recent advances in Wigner function approaches. Appl. Phys. Rev..

[7] Zurek WH (2003). Decoherence, einselection, and the quantum origins of the classical. Rev. Mod. Phys..

[8] Schlosshauer M (2005). Decoherence, the measurement problem, and interpretations of quantum mechanics. Rev. Mod. Phys..

[9] Hou Q, Yang W, Chen C, Yin Z (2016). Generation of macroscopic Schrödinger cat state in diamond mechanical resonator. Sci. Rep..

[10] Wright JC (2020). Schrödinger cat state spectroscopy—A new frontier for analytical chemistry. Anal. Chem..

[11] Castaños O, López-Saldívar JA (2012). Dynamics of Schrödinger cat states. J. Phys. Conf. Ser..

[12] Choi JR, Yeon KH (2008). Time-dependent Wigner distribution function employed in coherent Schrödinger cat states: $$\vert \psi (t) \rangle = {N}^{-1/2} ( \vert \alpha \rangle + e^{i\phi } \vert -\alpha \rangle )$$. Phys. Scr..

[13] Dodonov V, Dodonov A (2014). Transmission of correlated Gaussian packets through a delta-potential. J. Russ. Laser Res..

[14] Sokolovski D (2015). Interference effects in tunneling of Schrödinger cat wave-packet states. Phys. Rev. A.

[15] Gisin N, Thew R (2007). Quantum communicatios. Nat. Photon.

[16] Heller EJ (1999). The many faces of tunneling. J. Phys. Chem. A.

[17] Heller EJ (2018). The Semiclassical Way to Dynamics and Spectroscopy.

[18] Takabayasi T (1954). The formulation of quantum mechanics in terms of ensemble in phase space. Prog. Theor. Phys..

[19] Baker GA (1958). Formulation of quantum mechanics based on the quasi-probability distribution induced on phase space. Phys. Rev..

[20] Tatarskiĭ VI (1983). The Wigner representation of quantum mechanics. Sov. Phys. Usp..

[21] Schleich WP (2001). Quantum Optics in Phase Space.

[22] Dragoman D (2005). Phase space formulation of quantum mechanics. Insight into the measurement problem. Phys. Scr..

[23] Błaszak M, Domański Z (2012). Phase space quantum mechanics. Ann. Phys..

[24] Curtright TL, Fairlie DB, Zachos CK (2014). A Concise Treatise on Quantum Mechanics in Phase Space.

[25] Lozovik YE, Filinov AV (1999). Transmission times of wave packets tunneling through barriers. JETP.

[26] Kälbermann G (2001). Diffraction of wavepackets in space and time. J. Phys. A Math. Gen..

[27] Sokolovski D, Akhmatskaya E (2018). No time at the end of the tunnel. Commun. Phys..

[28] Petersen J, Pollak E (2018). Quantum coherence in the reflection of above barrier wavepackets. J. Chem. Phys..

[29] Bialynicki-Birula I, Rudnicki Ł (2011). Entropic Uncertainty Relations in Quantum Physics.

[30] Hertz A, Oreshkov O, Cerf NJ (2019). Multicopy uncertainty observable inducing a symplectic-invariant uncertainty relation in position and momentum phase space. Phys. Rev. A.

[31] Wigner E (1932). On the quantum correction for thermodynamic equilibrium. Phys. Rev..

[32] Hillery M, O’Connell RF, Scully MO, Wigner EP (1984). Distribution functions in physics: Fundamentals. Phys. Rep..

[33] Takahashi K (1986). Wigner and Husimi functions in quantum mechanics. J. Phys. Soc. Jpn..

[34] Lee H-W (1995). Theory and application of the quantum phase-space distribution functions. Phys. Rep..

[35] Benedict MG, Czirják A (1999). Wigner functions, squeezing properties, and slow decoherence of a mesoscopic superposition of two-level atoms. Phys. Rev. A.

[36] Kenfack A, Życzkowski K (2004). Negativity of the Wigner function as an indicator of non-classicality. J. Opt. B Quantum Semiclass. Opt..

[37] Sadeghi P, Khademi S, Nasiri S (2010). Nonclassicality indicator for the real phase-space distribution functions. Phys. Rev. A.

[38] Kenfack A (2016). Comment on “nonclassicality indicator for the real phase-space distribution functions”. Phys. Rev. A.

[39] Khademi S, Sadeghi P, Nasiri S (2016). Reply to “comment on ‘nonclassicality indicator for the real phase-space distribution functions’ ”. Phys. Rev. A.

[40] Moyal JE (1949). Quantum mechanics as a statistical theory. Proc. Camb. Philos. Soc..

[41] Hiley BJ (2015). On the relationship between the Wigner–Moyal approach and the quantum operator algebra of von Neumann. J. Comput. Electron..

[42] Deléglise S (2008). Reconstruction of non-classical cavity field states with snapshots of their decoherence. Nature.

[43] Mallet F (2011). Quantum state tomography of an itinerant squeezed microwave field. Phys. Rev. Lett..

[44] Jullien T, Roulleau P, Roche B, Cavanna YJ, Glatti D (2014). Quantum tomography of an electron. Nature.

[45] Ding S, Maslennikov G, Hablützel R, Loh H, Matsukevich D (2017). Quantum parametric oscillator with trapped ions. Phys. Rev. Lett..

[46] Tian Y (2018). Measurement of complete and continuous wigner functions for discrete atomic systems. Phys. Rev. A.

[47] Vanner MR, Hofer J, Cole GD, Aspelmeyer M (2013). Cooling-by-measurement and mechanical state tomography via pulsed optomechanics. Nat. Commun..

[48] Rashid M, Toroš M, Ulbricht H (2017). Wigner function reconstruction in levitated optomechanics. Quantum Meas. Quantum Metrol..

[49] Chen B (2019). Quantum state tomography of a single electron spin in diamond with Wigner function reconstruction. Appl. Phys. Lett..

[50] Groenewold HJ (1946). On the principles of elementary quantum mechanics. Physica.

[51] Curtright T, Uematsu T, Zachos C (2001). Generating all Wigner functions. J. Math. Phys..

[52] Bayen F, Flato M, Fronsdal C, Lichnerowicz A, Sternheimer D (1977). Quantum mechanics as a deformation of classical mechanics. Lett. Math. Phys..

[53] Bordemann M (2008). Deformation quantization: a survey. J. Phys. Conf. Ser..

[54] Kim K-Y (2007). A discrete formulation of the Wigner transport equation. J. Appl. Phys..

[55] Costolanski AS, Kelley CT (2010). Efficient solution of the Wigner–Poisson equations for modeling resonant tunneling diodes. IEEE Trans. Nanotechnol..

[56] Kim K-Y, Kim S (2015). Effect of uncertainty principle on the Wigner function-based simulation of quantum transport. Solid-State Electron..

[57] Muscato O, Wagner W (2016). A class of stochastic algorithms for the Wigner equation. SIAM J. Sci. Comput..

[58] Schulz D, Mahmood A (2016). Approximation of a phase space operator for the numerical solution of the Wigner equation. IEEE J. Quant. Electron..

[59] Thomann A, Borzì A (2017). Stability and accuracy of a pseudospectral scheme for the Wigner function equation. Numer. Methods Partial Differ. Equ..

[60] Feit MD, Fleck JA, Steiger A (1982). Solution of the Schrödinger equation by a spectral method. J. Chem. Phys..

[61] Torres-Vega G, Frederick JH (1991). Numerical method for the propagation of quantum-mechanical wave functions in phase space. Phys. Rev. Lett..

[62] Dattoli G, Giannessi L, Ottaviani PL, Torre A (1995). Split-operator technique and solution of Liouville propagation equations. Phys. Rev. E.

[63] Dattoli G, Giannessi L, Quattromini M, Torre A (1998). Symmetric decomposition of exponential operators and evolution problems. Physica D.

[64] Gómez EA, Thirumuruganandham SP, Santana A (2014). Split-operator technique for propagating phase space functions: Exploring chaotic, dissipative and relativistic dynamics. Comput. Phys. Commun..

[65] Cabrera R, Bondar DI, Jacobs K, Rabitz HA (2015). Efficient method to generate time evolution of the Wigner function for open quantum systems. Phys. Rev. A.

[66] Strang G (1968). On the construction and comparison of difference schemes. SIAM J. Numer. Anal..

[67] MacNamara S, Strang G, Glowinski R, Osher SJ, Yin W (2016). Operator splitting. Splitting Methods in Communication, Imaging, Science, and Engineering, Scientific Computation.

[68] Kołaczek D, Spisak BJ, Wołoszyn M (2019). The phase space approach to time evolution of quantum states in confined systems: The spectral split-operator method. Int. J. Appl. Math. Comput. Sci..

[69] Lee C-W, Jeong H (2011). Quantification of macroscopic quantum superpositions within phase space. Phys. Rev. Lett..

[70] Jeong H, Noh C, Bae S, Angelakis DG, Ralph TC (2014). Detecting the degree of macroscopic quantumness using an overlap measurement. J. Opt. Soc. Am. B.

[71] Feit MD, Fleck J, Steiger A (1982). Solution of the Schrödinger equation by a spectral method. J. Comput. Phys..

[72] Larkin KG, Oldfield MA, Klemm H (1997). Fast fourier method for the accurate rotation of sampled images. Opt. Commun..

[73] Myagotin A, Vlasov E (2014). Efficient implementation of the image rotation method using chirp z-transform. Pattern Recognit. Image Anal..

[74] Spisak BJ, Wołoszyn M (2009). Nonclassical properties of electronic states of aperiodic chains in a homogeneous electric field. Phys. Rev. B.

[75] Garbaczewski P (2005). Differential entropy and time. Entropy.

[76] Hertz A, Jabbour MG, Cerf NJ (2017). Entropy-power uncertainty relations: Towards a tight inequality for all Gaussian pure states. J. Phys. A.

[77] Wołoszyn M, Spisak BJ, Adamowski J, Wójcik P (2014). Magnetoresistance anomalies resulting from stark resonances in semiconductor nanowires with a constriction. J. Phys. Condens. Matter.

[78] Tannor D (2007). Introduction to Quantum Mechanics: A Time-Dependent Perspective.

[79] Maitra NT, Heller EJ (1996). Semiclassical perturbation approach to quantum reflection. Phys. Rev. A.

[80] Maitra NT, Heller EJ (1997). Barrier tunneling and reflection in the time and energy domains: The battle of the exponentials. Phys. Rev. Lett..

[81] Jaffe RL (2010). Reflection above the barrier as tunneling in momentum space. Am. J. Phys..

[82] Yamamoto M, Takada S, Böuerle C, Watanabe K, Wieck AD, Tarucha S (2011). Electrical control of a solid-state flying qubit. Nat. Nanotechnol..

[83] Kataoka M (2009). Coherent time evolution of a single-electron wave function. Phys. Rev. Lett..

[84] Yamahata G, Ryu S, Johnson N, Sim H-S, Fujiwara A, Kataoka M (2019). Picosecond coherent electron motion in a silicon single-electron source. Nat. Nanotechnol..

[85] Esin I, Romito A, Gefen Y (2020). Detection of quantum interference without an interference pattern. Phys. Rev. Lett..

